# Managing diabetes in the digital age

**DOI:** 10.1186/s40842-015-0016-2

**Published:** 2015-12-01

**Authors:** Viral N. Shah, Satish K. Garg

**Affiliations:** 1 Barbara Davis Center for Diabetes, University of Colorado Denver, 1775 Aurora Court, A140, Aurora, CO 80045 USA; 2 School of Medicine, University of Colorado Denver, Aurora, CO USA; 3 Diabetes Technology and Therapeutics, New Rochelle, USA

**Keywords:** Diabetes, Digital health, Artificial pancreas, Closed-loop system, Electronic health records, Mobile health, mHealth, Diabetes self-management, Mobile applications

## Abstract

The prevalence of diabetes is rising globally. Poor glucose control results in higher rates of diabetes-related complications and an increase in health care expenditure. Diabetes self-management education (DSME) training has shown to improve glucose control, and thus may reduce long-term complications. Implementation of diabetes self-management education programs may not be feasible for all the institutions or in developing countries due to lack of resources and higher costs associated with DSME training. With the increasing use of smartphones and Internet, there is an opportunity to use digital tools for training people with diabetes to self-manage their disease. A number of mobile applications, Internet portal, and websites are available to help patients to improve their diabetes care. However, the studies are limited to show its effectiveness and cost-benefits in diabetes self-management. In addition, there are many challenges ahead for the digital health industry. In this review, we assess the use of newer technologies and digital health in diabetes self-management with a focus on future directions and potential challenges.

## Introduction

The International Diabetes Federation (IDF) estimates a global epidemic of diabetes. In 2014, 387 million people had diabetes, and it will increase to 592 million by 2035 [1].

Poor glucose control leads to long-term diabetes, micro- and macro-vascular complications resulting in higher morbidity, and mortality that accounts for 4.9 million deaths in 2014 and $612 billion in health care expenditure [1]. Therefore, American Diabetes Association (ADA) recommends glycosylated hemoglobin (A1c) goal of 7 or less to prevent diabetes complications [2]. To achieve this goal, diabetes self-management education (DSME) is crucial [2, 3]. As expected, DSME is associated with a higher cost as $4.8 million were reimbursed by the Center for Medicare and Medicaid (CMS) in 2010 for DSME training for the Medicaid population [4]. In addition, about 77 % of people with diabetes live in low- and middle-income countries [1] that do not have adequate resources to provide such training to patients. Even in the USA, only about 20 % of adults with diabetes are cared for by an endocrinologist/diabetologist [5, 6].

The wireless broadband and smartphone market totals 1.5 billion globally as of 2013 which is expected to rise to 6.5 billion by 2018 [7]. With increasing numbers of smartphone users, it is possible to apply mobile app technology to empower patients to better manage their diabetes. As the number of people with diabetes rises, and with an inadequate number of specialists and lack of resources in developing countries, there is an opportunity and growing need to develop cost-effective supporting tools for DSME to improve overall diabetes outcomes.

In this manuscript, we review the use of newer technologies and digital health in diabetes self-management with a focus on future directions and potential challenges.

## Review

### Diabetes self-management goes digital

DSME and training is a collaborative process through which people with, or at risk for, diabetes gain the knowledge and skills needed to modify their behavior and successfully self-manage the disease to improve health outcomes [8]. The components of DSME are healthy eating, self-monitoring of blood sugar (SMBG), medication adherence, and diabetes complications risk reduction behavior. Table 1 summarizes the available mobile applications (apps) to help patients with diabetes to self-manage the disease. Management of diabetes is generally self-directed, and individuals need to make day-to-day decisions related to controlling their disease [8]. Effective management requires patients to understand and use appropriate technologies for glucose monitoring and medication compliance as well as complex treatment strategies; therefore, DSME is recognized as a crucial component in diabetes care and in limited pilot studies. It has been shown to be cost-effective and efficacious in lowering A1c and blood pressure [9–11].

**Table 1:** Mobile apps to support diabetes management^ab^

Diet	Physical activity	Blood glucose e-log book
Healthy out	Track 3	Diabetic
Foodily	My Fitness pal	Diabetes in check
Whole food market recipe	Moves	Diabetes companion
CarbControl	Nike + running	My sugar Junior
Lose it	Strava	Go meal
Weight watchers	UP by jawbone	Glooko
Daily burn	Endomondo	Glucose buddy
Calorie counter PRO	GymPact	DiabetesApp lite
iCookbook diabetic	FitnessFast	My net diary
Fooducate	Pacer	Glucose companion
EatLocal		
Calorie king		
HEALTHeDiabetes		
Glucose monitoring	Insulin dose calculators	Relaxation and meditation
iBGStar	Insulin calculator	Calm
Telcare	iBolus calc	Sleep cycle
	Insulin dose calculator pro	Equanimity
	Diabetes personal calculator	
Diabetes education	Rapid calc diabetes manager	Medication adherence
WebMD	PredictBGL	MyMedSchedule
Diabetes insight	EZ insulin calculator	MyMeds
Up to date	Insulin units	MedSimple
Managing type 1 Diabetes		Pillmanager
Diabetes EDC		Pill reminder
Diabetes @point of care		RxmindMe Prescription
		Pillboxie

^a^Most apps have more than one feature. ^b^the list is not comprehensive, there are number of apps available in each category

### Online diabetes education

The conventional education for diabetes self-management has been supplemented with several web portals, blogs, and structured online educational materials. The online portals and apps may be cost-effective, convenient, easy to use and learn anywhere at anytime to understand diabetes, its complications, and how to individualize and self-manage. Government organizations such as the National Institute of Diabetes and Digestive and Kidney Diseases (http://www.niddk.nih.gov/health-information/health-topics/Diabetes/Pages/default.aspx), professional diabetes organizations such as the ADA (http://www.diabetes.org) and the American Association of Clinical Endocrinologists (http://empoweryourhealth.org/endocrine-conditions/diabetes) and many pharmaceutical industries and other nonprofit organizations provides online information on diabetes, diabetes-related health problems and education. A number of apps such as Diabetes EDC, Point of Care Diabetes, Diabetes journal, Prognosis Diabetes, Diabetes Forecast, Diabetes Forum and Diabetes FAQ also provide basic diabetes education. In addition, researchers have also designed digital virtual environment [e.g. SLIDES (Second Life Impacts Diabetes Education and Self-management by Duke University, Durham, NC, USA] by creating virtual 3D community to provide DSME training based on social cognitive theory [12].

#### Healthy eating

Counting carbohydrates is useful for people with insulin-requiring diabetes to administer appropriate prandial insulin doses to maintain euglycemia [13, 14]. Pilot studies show that taking fat and protein into account for insulin dose calculations result in significant A1C reductions compared with only carbohydrate counting [15]. Similarly, counting calories and making healthy choices are equally important for weight management [16]. Several books on carbohydrate and calorie counting are available for the patients to use; however, it is inconvenient to carry books all the time. Therefore, apps help patients improve their nutritional choices and monitor their food and caloric intake. Many apps include a feature that allows users to search food databases by typing or scanning bar codes. For example, GoMeal, My Fitness pal, Calorie Counter by MyNetDiary, and Glucose Buddy offer extensive databases, allowing users to quickly look up nutritional information including carbohydrate content and calories. In addition, these apps offer target planning and goal setting to help users manage their weight and caloric intake. Above all, apps are in development to analyze food content based on images of the food [17].

#### Insulin dose calculators

Accurate bolus insulin doses require calculations based on factors such as current and target blood glucose, carbohydrate-to-insulin ratios, total grams of carbohydrate in meals, insulin sensitivity factors, and insulin on board [18]. It is difficult for insulin requiring patients to account for all these factors for their insulin dosing. Introduction of automatic bolus calculators integrated in the insulin pump (bolus wizard) have shown to help patients to more accurately meet prandial insulin dosage requirements, improve postprandial glycemic excursions, and achieve optimal glycemic control [18–20]. However, it was estimated that of 13.2 million people with diabetes, only 162,000 were insulin pump users in 2002 [21, 22]. The majority of people with diabetes do not use insulin pumps due to cost, lack of insurance coverage, or other unrelated issues. Apps such as Insulin Calculator, Bolus Calc, Insulin Dose Calculator Pro, and Diabetes Personal Calculator for non-pump users are available to help patients in insulin dosing. The Food and Drug Administration (FDA) has not approved most of the available apps.

#### Physical activity

The Surgeon General’s Report on Physical Activity and Health in 1996 highlighted the pivotal role of physical activity in health promotion and disease prevention [23]. Modest weight loss has been shown to improve insulin resistance in overweight and obese people with diabetes [24]. ADA recommends moderate weight loss (7 % of body weight) for overweight individuals with diabetes [2]. Key components of weight loss management are self-monitoring of physical activity by means of recording frequency, intensity, time, type of activity, and a healthy diet [25, 26]. However, recording physical activity puts an additional burden on participants. Therefore, many developers have created digital tools for physical activity monitoring such as pedometers, wristband sensors, and personal digital assistants. Examples of few mobile apps for monitoring steps and physical activities are; pacer, Steps pedometer and step counter activity tracker, Map My Walk, Stepz, Walker-Pedometer Lite, Footsteps, iRunner and Runtastic Pedometer. Fitbit, the Jawbone Up24 and the Nike Fuelband are examples of wristband sensor that tracks person’s physical activity. These apps allow users to track their activity, count calories, and provide ways for weight management. In addition, many apps help people change their physical activity by providing instruction to perform such activities, modeling/demonstrating, feedback on performance, planning social support/change, prompting review of goals, facilitating social comparison, setting graded tasks, and goal setting for a behavioral outcome [27].

Studies have shown that the use of apps results in greater weight loss compared to conventional physical activities without the help of tracking apps [28]. The Task Force for Community Preventive Services noted that health promotion activities tailored to an individual’s specific needs increase the likelihood of beginning an exercise program and increase the frequency of exercise [29]. It has also been noted that employee education programs for physical activity at the work place improved health outcomes [30]. Considering this, many organizations (e.g. Be Colorado Move program by the University of Colorado, http://becolorado.org/programs/be-colorado-move and EHP Wellness Program by Cleveland Clinic http://www.clevelandclinic.org/healthplan/wellness.htm) have initiated incentives for their employees to use tracking apps and record their daily physical activities. Studies have shown that financial incentives are more effective than usual care or no intervention for encouraging healthy behavior change [30–32].

#### Self-monitoring of blood glucose

Self-monitoring of blood glucose (SMBG) is recognized as an important tool for decision-making for both patients and health care providers [33]. ADA recommends that patients whose medication regimen includes multiple daily insulin injections or insulin pumps should test their blood glucose three times or more per day [2]. However, not all patients with diabetes test blood glucose three times or more a day. Hurdles in poor adherence to SMBG include a) inaccurate meters b) big and bulkier devices c) need to poke a finger multiple times d) need to carry an additional device all the time and e) paper log book entry [34, 35]. This results in poor compliance and inadequate glucose control with wide glucose excursions. With the advances in blood glucose meters and mobile technology, it has become possible to address several of these issues. For example, iBGStar is an external device that fits easily to an iPhone and functions as a glucose meter that helps patients carry their glucose meter along with a smartphone (http://www.ibgstar.us/). Studies have shown that iBGstar use is associated with higher patient satisfaction and better glycemic outcomes in adults with type 1 diabetes [36]. Most manufacturers have designed clinical decision supporting websites to download blood glucose meter to analyze the glycemic pattern and trends to help patients and clinicians with treatment decision. Example of such decision supporting tools are CareLink, LibreView and Accu-chek connect. A number of apps such as GoMeal, Diabetes Net Diary, and Glooko provide an e-Log book where blood glucose data can be saved and printed later or can be emailed to health care providers. In addition, these apps have graphical displays to see and interpret blood glucose entries and thus motivate patients to improve blood glucose testing frequency resulting in better glucose control. Most glucose meter manufacturers are now planning to integrate insulin calculators with traditional blood glucose meters to help patients on multiple daily injections take their bolus insulin dose. Recently, FDA approved Accu-Check Aviva Expert glucose meter with built-in insulin advisor as it has shown to improve glycemic control and treatment satisfaction without increasing severe hypoglycemia in insulin requiring patients with diabetes [37].

#### Medication adherence

Medication non-adherence remains a common health problem resulting in about 50 % of medication related hospitalization and accounts for about $100 billion in health care costs [38]. Medication non-adherence is very common in people with diabetes resulting in poor glycemic control [39, 40]. Studies have shown that short message service (SMS) results in better medication adherence and have opened avenues for apps development [41, 42]. The advantages of using the adherence apps are simple to use and navigate, data storage, medication instruction, and features to download and print a medication chart [41, 42]. Most of the medication adherence apps like MyMedSchedule, My Meds, MedSimple, Medagenda, Pillmanager, Pill reminder, and RxmindMe Prescription cost little.

### Digital health care information

The health care system is shifting to a value-based model. CMS launched the “electronic health record (EHR) financial incentive” program that mandates physicians to use EHR to document meaningful use [43]. With the increasing use of HER, which is Health Insurance Portability and Accountability Act (HIPAA) compliant, it has become possible to allow the patients to access their health records and communicate with the providers easily. It has been shown that patient access to personal health records has the potential to improve self-care and influence clinical decision-making. The large EHR systems in the USA: Kaiser Permanente (My Health manager), EPIC (My Chart), and VistA (U.S. Department of Veterans Affairs) have introduced an internet portal as well as apps to share health information with patients. Patient access of such systems has significantly reduced primary care office visits and telephone contacts [44]. Similarly, EMIS Access and Renal Patient View in the UK have been shown to reduce administration overload and secondary care [45].

### Outcomes with the use of digital tools for diabetes self-management

The use of digital health tools has increased. Almost one third of U.S. smartphone owners were using health apps in 2014, and half of them were using fitness-related apps [46]. Despite an increase in the use of health-related apps, data is lacking on its benefits and cost-effectiveness. Small studies have shown better glucose control, improved SMBG frequencies, better patient satisfaction, moderate weight loss, and medication adherence with the use of digital tools [17, 20, 28, 36, 41, 47]. A recent review of a major electronic database for clinical trials including 16 randomized controlled trials with 3,578 adult participants with type 2 diabetes mellitus by Pal and colleagues showed small benefits on glycemic control (pooled effect on HbA1c: −0.2 %) with the use of computer-based diabetes self-management interventions. In a subgroup analysis on mobile phone–based interventions, they found a larger effect on A1c reduction (pooled effect on HbA1c was −0.50 %). Nevertheless, they did not find benefits of computer- or mobile-based interventions on improving depression, quality of life, or weight. In addition, the authors highlighted deficiencies such as selection bias and inadequate randomization procedure [48].

In addition, studies from developing countries are promising for the use of mobile or internet technologies for diabetes management [49–51]. A large randomized control trial showed that type 2 diabetes could be prevented by changing lifestyles with frequent short messages (SMS) tailored to change subject’s behavior [49].

However, most of these studies are underpowered and of short duration. A recent large clinical trial randomized 151 patients with type 2 diabetes into a) the mobile phone-based self-management system [Few Touch Application (FTA); consists of blood glucose-measuring system with automatic wireless data transfer, diet manual, physical activity registration, and management of personal goals] b) FTA plus health counseling based on behavior change theory by a diabetes specialist nurse and c) control. The authors did not find a difference in HbA1c levels, self-management, health-related quality of life, depressive symptoms, or lifestyle changes between groups after the 1-year intervention [52]. In a review of 21 published studies from 2000 to 2010 by Holtz et al. that used mobile intervention for diabetes self-management, the authors concluded that most of these studies lacked a sufficient sample size or intervention length to determine whether the results are clinically meaningful. In addition, they also noted that the majority (95 %) of studies examined the use of mobile phones from a patient perspective [53]. Similarly, Baron and colleagues reviewed 20 published studies from 2002 to 2011 that investigated the clinical effectiveness of interventions requiring patients to transmit blood glucose (BG) readings to an online server via a mobile device. They concluded that evidences for effectiveness of mobile or online interventions for diabetes remains weak due to high variability between studies and methodological weaknesses [54].

### Barriers to use digital tools for diabetes management

1. *Cost:* A potential barrier to any new medical technology is the cost. In addition, the use of apps requires the person to use an expensive smartphone and an internet data plan. Most payers do not cover the cost of having these devices or apps due to lack of conclusive data. Though diabetes is a major problem in developing countries, these technologies have not been used due to the higher cost of smartphone devices and Internet services. In addition, internet access may be another big problem in certain rural part of developing countries as well as developed countries such as USA [55].
2. *Insufficient scientific evidences:* Despite increased enthusiasm with the use of digital tools for diabetes self-management, the evidence for safety, efficacy, and cost-effectiveness of these tools are largely unknown. As described earlier, most of the studies on effectiveness of apps or internet-based tools for diabetes self-management were underpowered to see a meaningful and statistical difference and were of short duration [48, 49]. In addition, source information available on the blogs or through social media that are not regulated may not be scientific and may mislead patients. Similarly, most of the nutrition and physical activity related mobile apps have not been evaluated for the accuracy of information or measures. Large randomized controlled trials of long duration are necessary to establish the safety, efficacy, and cost-effectiveness of apps in diabetes self-management.
3. *Not useful in certain populations:* Most available apps may not be useful for the elderly, non-English speakers, physically challenged, and subjects from a lower socio-economical status.
4. *Data protection:* With the increasing use of EHR, digital tools, smart watches, and apps, we generate a large amount of data that is stored on servers. There are a few problems with sensitive data storage by the institutions or governments wanting to store health records or national records. If the data is collected in one country and stored on the server in other country, a whole different set of legal rules might be enforced. In addition, there is a growing controversy on who owns the data: patients or the device or software owner?
5. *Data security:* Certain devices such as an artificial pancreas (e.g. insulin pump, CGM and blood glucose meter) are connected via Bluetooth. Wireless communication can be intercepted by electromagnetic devices or hacked by cyber attackers [56]. This poses significant risks to a person using such devices for diabetes management.
6. *Regulatory barriers:* Although the use of digital tools is helpful in the self-management of diabetes, improper use of digital tools or technical issues with the algorithms or software can lead to undesirable side effects. For example, insulin dose calculator software is helpful for patients with diabetes to determine bolus insulin doses. The technical problem can result in higher insulin dose calculation and can result in severe hypoglycemia. Considering the increasing use of digital health and its potential harms, the FDA issued a guideline for app and health software developers [57]. As recommended by the FDA, any computer- or software-based devices (including apps) intended to be used for the electronic transfer, storage, display, and/or format conversion of medical device data is considered a Medical Device Data Systems (MDDS) and they are classified in three different classes [Class III being high-risk to Class I being low-risk] based on the potential risks of using the software or a digital tool [57]. It has been recommended for the software or device developer to follow the regulatory requirements such as Establishment registration, Medical Device listing, Quality System (QS) regulation, Labeling requirements, Medical Device Reporting, and Reporting Corrections and Removals depending on the device or software risk [57]. Similarly, the European Union has also issued regulatory framework for the mHealh [58].

### Future of digital health for diabetes

Despite many barriers to overcome, the digital industry is growing at a fast pace. A $60 billion investment in digital health globally was made in 2013, and it is expected to reach $233 billion by 2020 [59]. Greater focus on digital health resulted in the U.S. government removing additional barriers for digital innovations (e.g. the introduction of the Healthcare Innovation and Marketplace Technologies Act [60]). Similarly, the FDA has also launched a “patient preference initiative” program to identify and develop methods for assessing patients’ benefits and risks related to specific conditions providing further boosts to the digital health industry [61].

Currently, EHR systems are not interconnected. However, in future, all EHR systems will hopefully be integrated using a common platform. Similarly, the developers of the mobile apps or digital devices are interested in interconnecting their devices with other platforms. For example, Fitbit integrates with various apps.

Recent attempts have been made to develop mobile software that can calculate nutritional information for the patients based on their food intake. Frøisland developed and tested a mobile-phone-based tool to capture (DiaMob) and visualize adolescents’ food intake aimed at understanding carbohydrate counting and to facilitate communication to daily treatment changes [17]. Implementing a visualization tool is an important contribution for young people to understand the basics of diabetes and to empower young people to define their treatment challenges. It empowers patients’ independence and management of their disease.

Insulin pumps and continuous glucose monitors have significantly changed the treatment outcomes in insulin-requiring diabetic patients. Studies have shown that insulin pump and CGM (sensor augmented therapy) result in better glucose control, less hypoglycemia, and reduce glucose excursion [62]. Two controllers or receivers overwhelm many patients; therefore, Medtronic and Animas insulin pumps have integrated CGM data on insulin pumps. Nevertheless, patients still have to wear CGM and insulin pumps separately. The research is under way to a) to prolong life of continuous glucose monitors b) integrate CGM with an infusion set (Pod Talk and Medtronic in-Duo) and c) replace finger stick blood glucose monitoring to non invasive ways of glucose monitoring. Similarly, artificial pancreas development is going to integrate algorithms in apps so that patients will be able to operate an insulin pump and CGM via mobile devices to have better patient acceptance and experience.

Recently, a growing interest in developing videogames to change health behaviors has arisen due to their increased popularity among adults and youth. Such games can change health behaviors by providing information on healthy food and physical activity [63].

Research is underway to develop apps to remotely monitor a patient’s health. For example, iExaminer is a small device that can be attached to an iPhone and take pictures of the retina, which can be transferred to the provider (http://www.welchallyn.com/en/microsites/iexaminer.html). Studies have shown that teleophthalmology is an effective model for improving eye care in underserved areas of developing countries [64].

## Conclusion

There is much enthusiasm amongst industry and patients to use digital tools for diabetes self-management. Large randomized control trials are needed to establish the effectiveness and cost-benefits of digital tools in improving diabetes-related outcomes. Despite many challenges to overcome, the future of the digital health industry is promising.
